# Intractable Hiccups in a Young Male: Is it a Tell-Tale Sign of Pseudocyst of Pancreas?

**DOI:** 10.7759/cureus.17951

**Published:** 2021-09-13

**Authors:** Dhruv Talwar, Sunil Kumar, Sourya Acharya, Sparsh Madaan, Vidyashree Hulkoti

**Affiliations:** 1 Medicine, Jawaharlal Nehru Medical College, Datta Meghe Institute of Medical Sciences (Deemed to be University), Wardha, IND; 2 Medicine, Jawaharlal Nehru Medical College, Datta Meghe Institute of Medical Science (Deemed to be University), Wardha, IND; 3 Obstetrics and Gynaecology, Datta Meghe Institute of Medical Science (Deemed to be University), Jawaharlal Nehru Medical College, Wardha, IND

**Keywords:** alcohol, chronic pancreatitis, pseudocyst of the pancreas, singultus, hiccup

## Abstract

Although encountered routinely in the outpatient department, hiccups or singultus are mostly neglected by the clinicians owing to its benign presentation and self-limiting nature. However, an innocent-looking symptom such as a hiccup can be a warning sign of serious underlying medical conditions and hence should be addressed seriously. Hiccups can seriously alter the quality of life and induce anxiety when they become intractable. We present an atypical case of a 30-year-old male who presented with intractable hiccups for four months and upon investigations revealed to be a case of chronic pancreatitis with pseudocyst of the pancreas. The patient's intractable hiccups were not responding to metoclopramide and responded well to gabapentin. The patient was managed conservatively for chronic pancreatitis and pseudocyst of the pancreas. This case report highlights the importance of investigating the cause of a simple symptom like hiccup as it can be a tell-tale sign of a chronic underlying pathology such as pseudocyst of the pancreas in our case. After an extensive review of literature, it was found that this is the first case to report intractable singultus as a result of the pseudocyst of the pancreas.

## Introduction

Hiccups are a familiar symptom encountered by all clinicians, however, it remains a poorly understood phenomenon that includes involuntary contraction of the diaphragm which is repetitive and may also involve the intercostal muscles [1]. The medical term for hiccup is "singultus" which is derived from Latin translating to act of sobbing. The contraction of inspiratory muscles leads to rapid sudden inspiration of air which is interrupted by closure of the glottis producing the characteristic "hic" sound of the hiccup. Its frequency may vary from 4 to 60 times in a minute [2]. In adults, hiccups do not seem to serve any physiological purpose; however, in the in uteros life hiccups are believed to train the fetal inspiratory muscles for respiration after delivery. Singultus is classified based on duration. It can be acute if lasting for less than 48 hours. Persistent singultus lasts for a duration of more than 48 h and intractable singultus lasts for a duration of more than one month [3]. Acute singultus is usually self-limiting and rarely requires medical intervention as its course is shortened with various maneuvers. Hiccups are commonly associated with gastrointestinal or central nervous system disorders. While some common gastrointestinal causes of singultus such as diaphragmatic hernia, esophageal carcinoma, esophageal ulcer, and reflux esophagitis are described in various studies, a disease affecting the pancreas is a rare cause for intractable singultus [4]. Although diseases such as pancreatic carcinoma have been reported in rare cases to be presenting with singultus, pseudocyst of the pancreas has never been reported to be associated with intractable singultus making this an extremely rare presentation. In this case report, we present a case of a 30-year-old male who presented with intractable singultus for four months which upon investigations revealed to be as a result of pseudocyst of the pancreas associated with chronic pancreatitis.

## Case presentation

**Figure 1:**
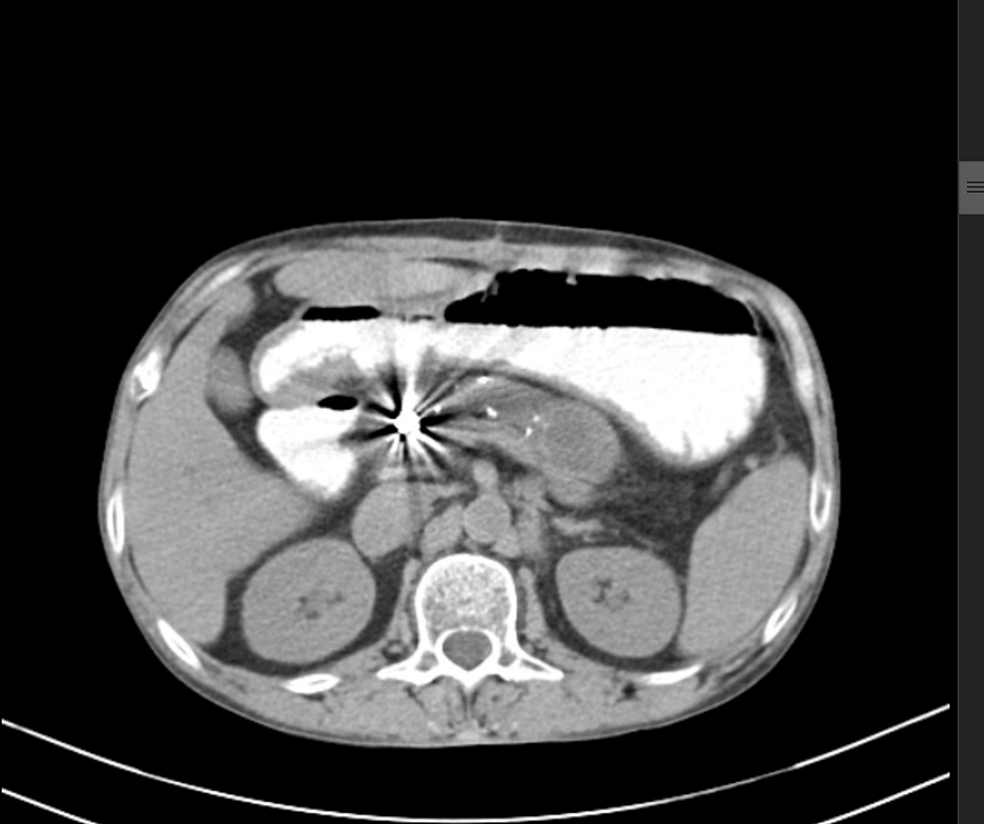
CECT abdomen showing chronic pancreatitis with calcification and pseudocyst involving the tail of pancreas. CECT, contrast-enhanced computed tomography of the abdomen

A 30-year-old male presented to the outpatient department with the chief complaint of intractable hiccups for four months. There was a history of intermittent pain in the epigastrium for four months which was dull in nature and non-radiating. The patient had no history of nausea, vomiting, or loose stools. There was no history of jaundice. There was no history of fever. The patient had history of chronic alcohol abuse with the last intake six months back with a CAGE score (Cut down, Annoyed, Guilty, and Eye-opener) of two out of four. He was admitted two months back when he was diagnosed as a case of chronic pancreatitis on the basis of a CT scan and a gastroscopy was done which did not show any significant finding followed by an endoscopic retrograde cholangiopancreatography (ERCP) procedure revealing biliary stricture which was treated by a prosthesis placement. The patient had no history of hypertension, diabetes mellitus, or any other chronic illness in the past. On general examination, the patient's pulse was 98 beats per minute, regular in rhythm, blood pressure was 110/70 mmHg in right arm supine position, pallor was present, and spo2 was 98 percent on room air with a respiratory rate of 24 breaths/minute. On systemic examination the patient had tenderness in the epigastrium, the heart sounds were normal, the chest was bilaterally clear, and the patient was conscious and oriented. Lab investigations revealed a normal electrolyte panel with normal liver and renal function tests. Serum amylase was 42 units/L and serum lipase was 46 units/L. A contrast-enhanced CT scan of the abdomen was carried out which was suggestive of chronic pancreatitis with pseudocyst of pancreas involving the tail of the pancreas (Figure 1). The patient was initially started on metoclopramide; however, he did not respond well and was then started on tablet gabapentin 100 mg BD with which there was a dramatic improvement in singultus. The patient was provided with creon 72,000 lipase units per meal (according to standard guidelines) because of exocrine pancreatic insufficiency diagnosed through fecal elastase test with fecal elastase measured as 75 μg/g feces. The patient was ultimately discharged after six days of admission in stable condition and is doing well on follow-up.

## Discussion

**Figure 2:**
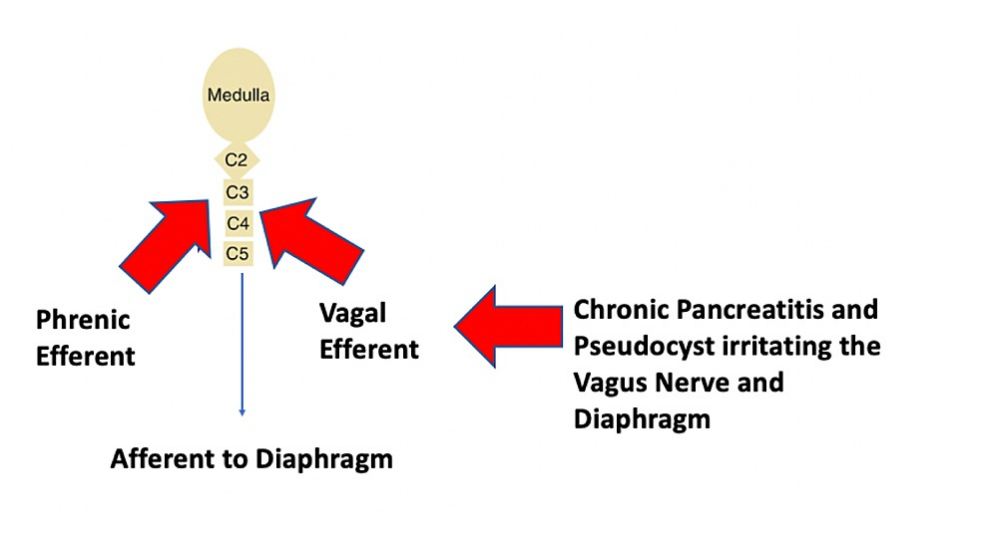
Pathophysiology of singultus in our case.

Hiccups are a result of myoclonic contractions of the diaphragm which are spontaneous and may also involve the intercostal muscles. In 1943 Bailey stated that hiccups are generated through a reflex arc comprising of an afferent, central, and efferent component. The afferent impulse is carried through the phrenic nerve, vagus nerve, or sympathetic nerve fibers with thoracic outflow from T6 to T12. Areas of the central nervous system which are involved in hiccups are situated in the upper part of the spinal cord (from C3 to C5), the brainstem in the medulla oblongata which is near the respiratory center, the reticular formation and hypothalamus. Neurotransmitters that modulate this mechanism are dopaminergic and gamma-amino-butyric-acid neurotransmitters. The efferent arc of this reflex is formed through the phrenic nerve which supplies the diaphragm that leads to contraction unilaterally or less commonly bilaterally. Activation of accessory nerves leads to the contraction of intercostal muscles. The events of reflex are completed by reflex closure of the glottis through the recurrent laryngeal branch of the vagus nerve [5]. The closure of the glottis is a protective mechanism because without it in patients with tracheotomy, hiccups lead to hyperventilation. The causes of hiccups thus can be any process that alters this reflex arc. The most common cause of hiccups is dilatation of the stomach following a large meal. It can also be triggered through alcohol, chilli pepper, smoking, and other irritants of the gastrointestinal tract and pulmonary tract. Anxiety-induced aerophagia can also lead to bouts of hiccups. However, patients with intractable singultus or hiccups should be investigated for organic causes involving various organ systems. Central nervous system causes can include vascular causes such as ischemic or hemorrhagic insult, infectious causes including meningitis and encephalitis, and structural causes such as brain injury and intracranial tumor. Other causes affecting the central nervous system leading to singultus include neuromyelitis optica, Parkinson's syndrome, epilepsy, and multiple sclerosis. Gastrointestinal causes of intractable singultus include gastroesophageal reflux disease, hiatus hernia, esophageal carcinoma, distention of the stomach, peptic ulcer, abdominal abscess, abdominal tumor, and pancreatitis. Thoracic causes include myocardial infarction and bronchial carcinoma while metabolic causes include hyponatremia, hypokalemia, and hypocalcemia. Psychological causes include anxiety, stress, and phobia [6]. Investigations of intractable singultus include investigating the entire reflex arc starting from a thorough history taking about drug intake, alcohol and smoking. The physical examination should include examination of ear, nose and throat, plus chest auscultation, and detailed abdominal examination. Blood investigations should be carried out to rule out electrolyte abnormalities and imaging modalities such as CT scan of the head or abdomen to look for the structures involved in the reflex arc. If no pathology is found for intractable singultus esophageal manometry and 24-hour pH impedance reflux study is indicated as gastroesophageal reflux disease is the most common cause of singultus. An endoscopy might be performed to look for other gastrointestinal causes such as a peptic ulcer. Treatment modalities for singultus include nasopharyngeal stimulation via intranasal application of vinegar or smelling of ammonia and oropharyngeal stimulation through ice water. Vagal stimulation such as cold compress to the face, carotid massage, and induced fright can also disrupt the hiccup arc relieving the patient of singultus. Respiratory maneuvers such as breath holding and rebreathing induced hypercapnia along with the valsalva maneuver can also be used to treat hiccups. Pharmacological treatment comprises metoclopramide and most recently drugs such as gabapentin are being used to treat hiccups [7]. In cases of intractable hiccups that fail to respond to drug therapy, a variety of invasive procedures have been used. These include peripheral anesthetic blocks to nerves involved in the singultus 'reflex arc', surgical disruption or stimulation of vagal afferents, or phrenic efferent nerves. In our case, the patient had not consumed alcohol for six months ruling it out as a cause of singultus. On physical examination ear nose throat and chest auscultation was normal along with normal central nervous system examination thereby ruling out pulmonary and central nervous system causes. There was mild tenderness present on abdomen examination in the epigastrium along with pseudocyst of the pancreas associated with chronic pancreatitis revealed on CT scan of the abdomen making it a most likely cause of intractable singultus in our case. This pseudocyst of pancreas was detected for the first time during this admission and was treated conservatively. We postulate that there might be irritation of the diaphragm and the vagus or phrenic nerve due to fluid collection seen in lesser sac because of pseudocyst of the pancreas leading to hiccups. Another school of thought is that pancreas is supplied by the vagus nerve which might be irritated by inflammation and fluid collection leading to hiccups in our case (Figure 2). This was treated with tablet gabapentin with conservative pharmacological management. Therefore this case report highlights the importance of underlying chronic conditions for a simple symptom such as singultus which should not be ignored by the treating physicians.

## Conclusions

Singultus which is intractable can be debilitating for patients and is often a neglected complaint in the outpatient department. We conclude that a proper workup should be performed for even a simple presenting symptom such as singultus which might be a tell-tale sign of underlying chronic conditions such as pseudocyst of the pancreas in our case thereby leading to prompt treatment and relief of intractable singultus.
